# Supplementary material for: Hypergamy reconsidered: Marriage in England, 1837–2021
Source: PLoS One. 2025 Feb 3;20(2):e0316769. doi: 10.1371/journal.pone.0316769 (PMC11790178; doi:10.1371/journal.pone.0316769)
Supplement: S1 File — (PDF) [file pone.0316769.s001.pdf]

Supplementary Material for *Hypergamy Reconsidered: Marriage in England, 1837-2021**

Gregory Clark and Neil Cummins*

December 31, 2024

1 Summary Statistics

Figure S1 plots the coordinates of each of the 5,184 parishes from which the sample's 1.7m marriage records were originally sourced. Table S1 reports the summary statistics for the church marriage database, 1837-2021.

Table S1: Summary Statistics, Parish Marriage Registers, 1837-2021

Statistic	N	Mean	St. Dev.	Min	Median	Max
Year	1,668,713	1,880.25	31.92	1,837	1,875	2,021
Groom Rank	1,441,596	33.61	15.36	0.00	33.90	100.00
Father Rank	1,338,504	33.75	15.88	0.00	33.31	100.00
Father-in-law Rank	1,337,912	33.15	15.87	0.00	33.31	100.00

*Gregory Clark; University of Southern Denmark, LSE and CEPR. Neil Cummins; LSE and CEPR.

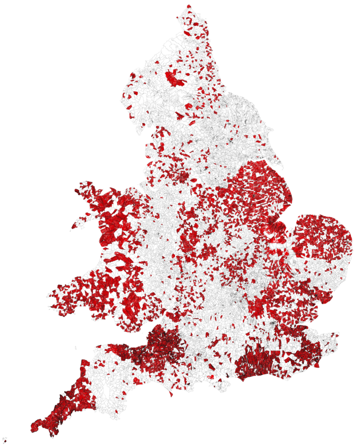

Figure S1: Map of the 5,184 Parishes in the Marriage Register Data

2 Tables of Results

This section presents tables of the results reported in the main paper's figures. Table S2 reports the mean status difference, and 95% confidence interval, between fathers of grooms and fathers of brides, by decade, 1837-2021 (this corresponds to figure 2 in the main paper). Table S3 reports the mean surname status difference, and 95% confidence interval, between grooms and brides, by decade, 1912-2007 (this corresponds to figure 3(a) in the main paper). Table S4 reports the mean surname status difference, and 95% confidence interval, between mother and fathers, by decade, 1912-2007 (this corresponds to figure 3(b) in the main paper).

Table S5 mean father occupational status for each partner in marriage by decile of the first partner's father's status (this corresponds to figure 6(b) in the main paper). It thus reflects the strength of assortment by men and women in marriage. Table S6 reports the mean surname status for each partner in marriage by decile of the first partner's surname status 1912-2007 (this corresponds to figure 6(c) in the main paper). Table S7 reports the mean surname status for each parent of a child by decile of the first partner's surname status 1912-2007 (this corresponds to figure 6(d) in the main paper).

Table S2: Father Differences in Status at Marriage, by Period

		Avg. Occupational Status			
Period	N	Groom's Father	Bride's Father	Diff.	se
1837-59	395,682	32.31	32.05	0.258	0.000
1860-99	544,543	33.26	32.52	0.742	0.000
1900-39	257,997	34.63	33.69	0.943	0.000
1940-79	45,935	41.61	41.22	0.387	0.000
1980-2021	11,871	52.05	52.35	-0.301	0.002

Source: Marriage Database, All Marriages, 1837-2021

Table S3: Spouse Surname Status Difference Means, and 95% Confidence Intervals, by Decade, 1910-2000

Decade	Mean Spouse Surname Status Difference	Confidence Interval Lower	Confidence Interval Upper
1910	0.004	0.002	0.007
1920	0.005	0.003	0.007
1930	0.005	0.003	0.007
1940	0.005	0.004	0.007
1950	0.002	0.000	0.004
1960	0.003	0.001	0.005
1970	0.003	0.002	0.005
1980	−0.001	−0.003	0.001
1990	−0.002	−0.004	−0.000
2000	−0.001	−0.004	0.001

Source: Universe of Marriages, 1912-2007.

Table S4: Parents Surname Status Difference Means, and 95% Confidence Intervals, by Decade, 1912-2007

Decade	Status Father	Status Mother	Mean Parent Surname Status Difference	Confidence Interval Lower	Confidence Interval Upper	N
1910	11.776	11.770	0.006	0.003	0.008	66,308
1920	11.772	11.769	0.004	0.002	0.006	80,960
1930	11.770	11.767	0.003	0.001	0.005	69,119
1940	11.775	11.769	0.006	0.004	0.008	81,578
1950	11.773	11.771	0.002	−0.000	0.004	85,298
1960	11.774	11.771	0.002	0.000	0.004	98,795
1970	11.779	11.772	0.007	0.005	0.009	78,687
1980	11.775	11.767	0.009	0.007	0.011	73,364
1990	11.779	11.769	0.010	0.008	0.013	75,405
2000	11.789	11.783	0.006	0.003	0.008	56,165

Source: Universe of Births, 1912-2007.

Table S5: Spouse Status Differences, Means, by Gender, 1837-1899

Decile	Men Status	Men Spouse Status	Women Status	Women Spouse Status
1	1.603	17.892	1.565	18.830
2	16.487	23.473	16.426	24.655
3	21.096	26.316	21.087	27.046
4	34.787	33.113	34.748	33.766
5	44.044	37.154	44.199	38.287
6	52.361	41.423	52.374	42.192
7	62.872	47.664	62.771	48.847
8	74.325	58.946	74.361	60.909
9	83.731	68.956	83.757	71.204
10	97.476	84.045	97.551	83.743

Source: Marriage Database, 1837-1899

Table S6: Spouse Surname Status Differences, Means, by Gender, 1980-2007

Decile	Men Status	Men Spouse Status	Women Status	Women Spouse Status
1	11.307	11.587	11.298	11.590
2	11.509	11.688	11.510	11.684
3	11.605	11.719	11.605	11.720
4	11.682	11.751	11.683	11.749
5	11.749	11.771	11.750	11.770
6	11.810	11.794	11.813	11.791
7	11.867	11.809	11.870	11.808
8	11.925	11.828	11.929	11.826
9	12.009	11.855	12.016	11.852
10	12.255	11.936	12.259	11.933

Source: Universe of Marriages, 1912-2007.

Table S7: Co-Parent Surname Status Differences, Means, by Gender, 1980-2007

	Men		Women	
Decile	Status	Co-Parent Status	Status	Co-Parent Status
1	11.314	11.604	11.275	11.604
2	11.513	11.690	11.501	11.690
3	11.610	11.713	11.601	11.713
4	11.688	11.747	11.681	11.747
5	11.756	11.770	11.750	11.770
6	11.818	11.792	11.814	11.792
7	11.873	11.799	11.871	11.799
8	11.932	11.821	11.931	11.821
9	12.018	11.847	12.020	11.847
10	12.273	11.933	12.266	11.933

Source: Universe of Births, 1912-2007.

3 An Alternative Surname Status Measure: Period Specific Wealth-at-Death

In this section we replicate the hypergamy analysis at the surname level with an alternative measure of surname status. In the main paper we use post-code averaged house values from the land registry 1995-2005, assigned to individuals whose address we observe in the 1999 electoral roll.

Though we measure surname status 1912-2007 using house values in 1999 from the electoral register house addresses, house value in fact correlates well with occupational status for men where we observe both their occupational status and their electoral register address 1999 and later. Figure S2 shows the regression link between house value post 1999 and occupational status for a sample of 1,657 men, mostly born before 1925, using the CCC occupational status index reported in Clark et al. (2024).

Here we instead use period specific wealth-at-death values observed at the individual level from a complete digitization of the Principal Probate Registry Calendars. This data and its characteristics are detailed extensively in a set of publications (Cummins (2021, 2022b); Cummins and Ó Gráda (2022); Cummins (2022a)). We average these individual level observations across surnames, separately for three periods; 1910-39, 1940-79, and 1980-1992. Table S8 reports the correlations between each period for surname status.

Table S8: Surname Status Correlations across Periods

	Surname Wealth		
	1940-39	1980-92	1980-92
	(1)	(2)	(3)
Surname Wealth$_{1910-79}$	.331***	.209***	
	(.004)	(.011)	
Surname Wealth$_{1940-79}$			.449***
			(.015)
Observations	19,180	9,037	10,393
R^2	.234	.040	.077

Note: *p<0.05; **p<0.01; ***p<0.001
OLS
Surname Count Range 10-500
(Based on 1999 Electoral Roll)

Figure S3 replicates figure 3(a) of the main paper, reporting the average percentage difference in status between the surnames of grooms and brides in marriages in England, by decades, from the 1912 to 2007. The results are consistent with those reported using the other surname status measure.

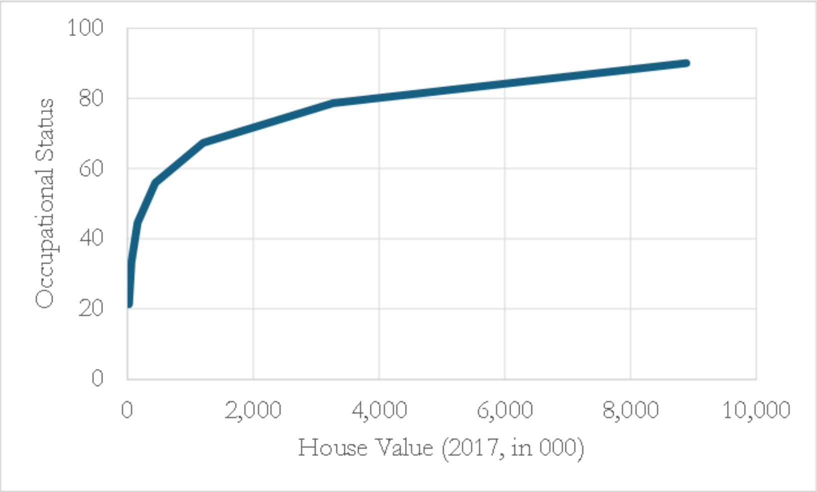

Figure S2: Occupational Status and Post-Code House Value of Surname
Notes: Occupational status is measured using the CCC occupational status index in Clark et al. (2024). The index has values 0-100.

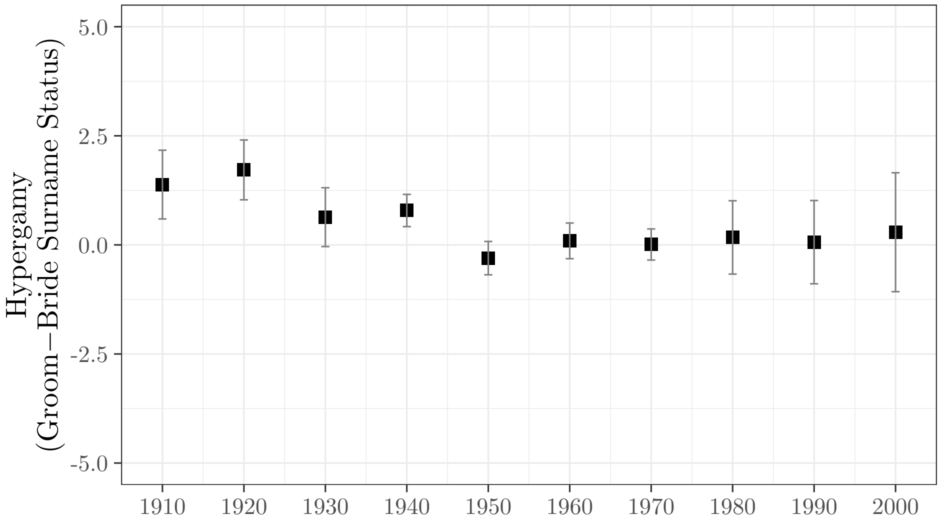

Figure S3: Status Differences in Marriage in England, Universe of Marriages 1912-2007, Alternative Surname Status Measure
Notes: We use the complete individual records of the Principal Probate Registry to average wealth-at-death across surnames for 1910-39, 1940-79, and 1980-1992 (for this last period we apply those wealth values to marriages 1980-2007). We drop surnames where we have less than 5 observations of probates within a period. The y-axis measures the difference in log values of wealth between spouses at marriage and therefore can be read as a percentage difference.

4 Hypergamy and Age at Marriage

How does age interact with hypergamy? Are our results robust to including age difference as a control variable? Do we observe hypergamy for younger brides and older grooms? In our data, most marry someone close in age and in status (see figure S4 (a) but the age difference distribution is skewed towards many more older grooms at marriage than older brides (figure S4 (b)).

We run a regression of the form

$$h = \alpha + \beta A_{g-b} + \varepsilon \tag{1}$$

where h is the status difference at marriage (groom $-$ bride status, as measured by the occupational score of their fathers), the subscripts b and g denote bride and groom respectively, A is age difference at marriage, α is a constant term, and ε an error. Table S9 reports the results of this regression for the periods used in the main paper. From 1837-1939 we find a consistent and positive but very modest correlation between a positive age gap (an older groom), and the status gap (a higher status groom). However the scale of this effect is very modest. There is some evidence for this effect disappearing by the latter half of the 20th century, as reveled by the diminution of the Age Difference coefficient in 1940-79, and it's reversal in 1980-2021.

In figure S5we report the relationship between age difference at marriage and hypergamy for the church data.

Table S9: Hypergamy and Age Difference at Marriage, by Period

	Status Difference in Marriage					
	1837-2021	1837-59	1860-99	1900-39	1940-79	1980-2021
	(1)	(2)	(3)	(4)	(5)	(6)
Age Difference	.074***	.077***	.076***	.088***	.035	−.211***
	(.004)	(.009)	(.005)	(.007)	(.021)	(.040)
Observations	868,309	127,899	429,205	253,792	45,581	11,832
R^2	.0005	.001	.001	.001	.0001	.002

Note: *p<0.05; **p<0.01; ***p<0.001
OLS

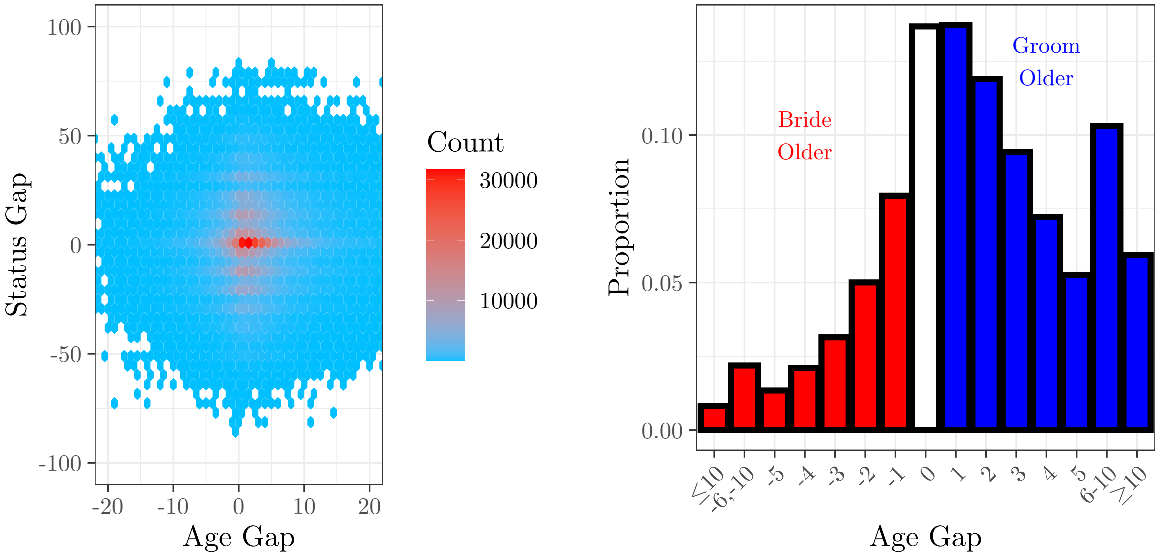

(a) Heat-map

(b) Distributions of Age Gaps at Marriage

Figure S4: Age Difference at Marriage, Distributional Characteristics
Note: The Hypergamy score is calculated as the occupational status (scored on a 0-100 scale) difference at marriage of the fathers of the bride and groom, and Age Gap at marriage as groom age minus bride age (in integer values as reported on the marriage certificates).

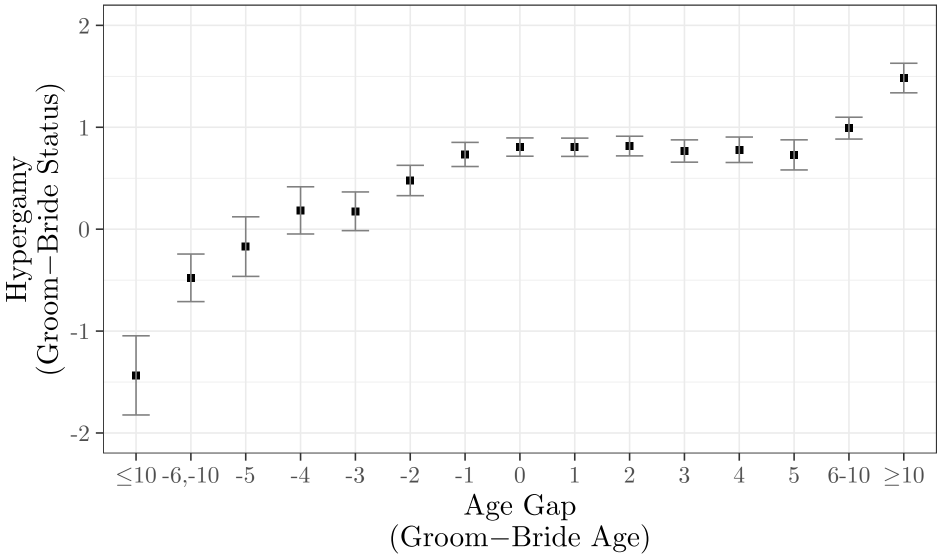

Figure S5: Age Difference at Marriage and Hypergamy
Note: The Hypergamy score is calculated as the occupational status (scored on a 0-100 scale) difference at marriage of the fathers of the bride and groom, and Age Gap at marriage as groom age minus bride age (in integer values as reported on the marriage certificates).

5 Groom Age and Occupational Status

Perhaps hypergamy exists because women marry men who at the time of marriage have no higher status than their fathers, but who will over the course of their careers gain much greater occupational status. Using the church marriage data we do see for marriages 1837-69 that older grooms have on average higher status, as is shown in figure S6 (dotted line). However, also in the figure we also plot occupational status of grooms by age controlling for father occupational status. Now the rise in occupational status of grooms with age is seen to mainly come from grooms from lower status families marrying earlier. Controlling for father status there is little rise in groom status with age. Groom status at marriage is close to expected groom status at age 40 or later. There is no evidence for the suggested mechanism for hypergamy.

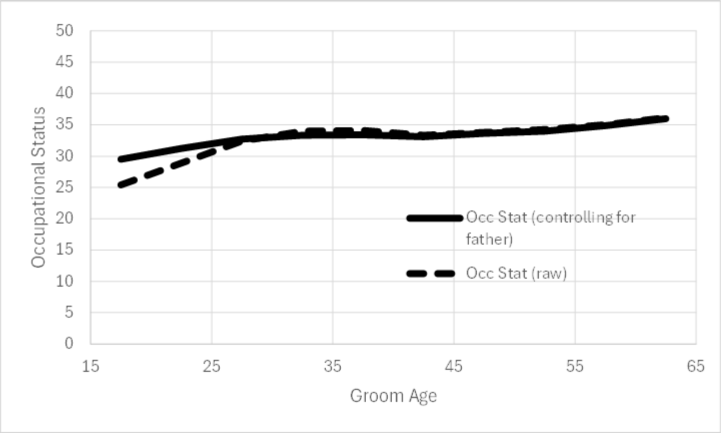

Figure S6: Groom occupational status 1837-69 by age, and controlling for father occupational status
Notes: Occupational status is measured using the CCC occupational status index in Clark et al. (2024). The index has values 0-100.

References

Clark, Gregory, Neil Cummins, and Matthew Curtis, "Three new occupational status indices for England and Wales, 1800–1939," *Historical Methods: A Journal of Quantitative and Interdisciplinary History*, 2024, *57* (1), 41–66.

Cummins, Neil, "Where Is the Middle Class? Evidence from 60 Million English Death and Probate Records, 1892–1992," *The Journal of Economic History*, 2021, *81* (2), 359–404.

_ , "Ethnic Wealth Inequality in England and Wales, 1858-2018," 2022.

_ , "The hidden wealth of English dynasties, 1892–2016," *The Economic History Review*, 2022, *forthcoming.*

_ **and Cormac Ó Gráda**, "The Irish in England," *Mimeo*, 2022.
