# Supplementary material for: Hypergamy reconsidered: Marriage in England, 1837–2021
Source: PLoS One. 2025 Feb 3;20(2):e0316769. doi: 10.1371/journal.pone.0316769 (PMC11790178; doi:10.1371/journal.pone.0316769)
Supplement: Attachment — Submitted filename: Responses to Editor Comments (2).docx [file pone.0316769.s002.docx]

**Responses to Editor Comments/Suggestions**

**- The findings are largely dependent on the claim that the factor of age from the data is a proxy for physical attractiveness, the source of which is not referenced by the literature nor made clear. This claim appears throughout the manuscript, for example: "For women, age can be taken as a correlate of physical appearance, with younger women more physically attractive in the marriage market", which is not supported by any academic references. We feel that this claim should be made much more explicit in the Introduction (and throughout).**

**Two responses on this.**

(1) **Only 1 out of 5 figures which present the main results of the paper depend on using age as a correlate of female physical attractiveness. The other 4 figures show the absence of female hypergamy in England 1837-2021 without any reliance on age being a proxy for female physical attractiveness.**
(2) **We now include in the text a brief summary of 3 recent scholarly papers which show that female age is indeed a correlate of physical attractiveness in the settings of speed dating and online dating. This is:**

Supporting this, a study looked at speed dating, where men and women with no prior information about each other, conversed for 3 minutes, before choosing whether they desired further contact. The major factors determining positive choices were all physical, for both women and men. Women with lower BMI, younger age, and greater self-rated facial attractiveness generated more positive responses. Women’s height had no effect. For women age and positive partner responses had a highly statistically significant negative correlation.^7^ Similarly a study of online dating behavior showed that, controlling for social status, there was a strong revealed preference by men for younger women. In contrast women had a weak positive preference for older men.^8^ Consistent with this, male ratings of the attractiveness of female faces declined more with age than did female ratings of the attractiveness of male faces.^9^

^7^Kurzban, Robert, and Jason Weeden, HurryDate: Mate preferences in action, *Evolution and Human Behavior 26(3)*, 2005: 227-244. https://doi.org/10.1016/j.evolhumbehav.2004.08.012

^8^Bruch, Elizabeth E. and M. E. J. Newman. Aspirational pursuit of mates in online dating markets. *Science Advances* **4**, 2018, eaap9815. DOI:[10.1126/sciadv.aap9815](https://doi.org/10.1126/sciadv.aap9815)

^9^Ebner, N. C. Age of face matters: Age-group differences in ratings of young and old faces. *Behavior Research Methods*, 40(1), 2008, 130–136.

**- In the Methodology, you use CAMSIS 1990 scores of social status for marriages 1940-2021. For marriage 1837-1939 you have constructed their own occupational status association index. While you have responded partly to reviewers query about this, we don't feel the submission fully explains why two different indexes were used and why the cut-off of 1939/1940 was chosen.**

**In the text we have added the clarification:**

We employ a separate occupational status index for 1837-1939 because over the long interval 1837-2021 many new occupations emerged, and the social status of some occupations changed significantly. Thus the 1837-1939 index shows a much higher father-son correlation of 0.71 1837-1879, compared to only 0.58 for the CAMSIS 1990 index for that period. Figure 2, showing an absence of significant hypergamy 1837-1939, would be unchanged if we instead measured father status 1837-1939 using the CAMSIS 1990 index.

We used the earlier occupational status index for 1837-1939 because we anticipated being criticized for using an inappropriate status index for the earlier years, had we used CAMSIS 1990 throughout. We chose the cutoff of 1940 because the period 1940-2021 saw much faster rates of technological change and economic growth than 1837-1939.

**- Reviewer 1 commented on how fathers' social status may not be the same as their children's social status, and further exploration of parental status vs child status vs married status would be helpful, and while your response to this was considered, we feel this limitation should be at least explicitly stated.**

**- Additional consideration and discussion of the role of maternal status would be helpful**

**In the paper we measure status by indicators of family status, either fathers’ occupational status, or surname status. We do this because for most of the period covered, women do not have a status of their own, but only their family status – birth family or husband. This provides a concrete and consistent measure of hypergamy: what is the family status at birth of women relative to their husband’s family status at birth?**

**Son and daughter status will regress to the mean compared to fathers’ status. But if that regression to the mean is the same for sons as for daughters, then measuring status using father status will not bias our findings. The only way this indirect measure of child status would be misleading would be if daughter status systematically declined relative to father status. We see no reason to expect this.**

**But to try and address this referee concern in the paper we have added the following paragraph in the discussion section.**

As noted above, we consistently measure hypergamy relative to birth-family status of husband and wife. If daughters’ attained status systematically declined to relative birth-family social status, in a way that sons’ status did not, then there would be a form of hypergamy. But we find no evidence that brides in England 1837-2021 were regarded as having degraded status relative to their birth families.

**- Finally, the structure of the submission also remains unclear, and while the authors have made some changes related to this, we kindly request a clearer adherence to the PLOS ONE manuscript guidelines (**[**https://journals.plos.org/plosone/s/submission-guidelines#loc-manuscript-organization**](https://journals.plos.org/plosone/s/submission-guidelines#loc-manuscript-organization)**).**

**The manuscript has been re-arranged using recent PLOS published papers also as a guide.**

**We have strictly conformed to the suggested pattern of**

**Abstract**

**Introduction**

**Methods**

**Results**

**Discussion**

**Acknowledgements**

**References**
